# Some properties of subclass of multivalent functions associated with a generalized differential operator

**DOI:** 10.1038/s41598-024-58781-6

**Published:** 2024-04-16

**Authors:** Haewon Byeon, Manivannan Balamurugan, T. Stalin, Vediyappan Govindan, Junaid Ahmad, Walid Emam

**Affiliations:** 1 Department of AI and Software, Inje University, Gimhae, 50834 South Korea; 2 Department of Mathematics, Vel Tech Rangarajan Dr.Sagunthala R&D Institute of Science and Technology, Chennai, India; 3 Department of Mathematics, Hindustan Institute of Technology and Science, Chennai, India; 4 Department of Mathematics and Statistics, International Islamic University, H-10, Islamabad, 44000 Pakistan; 5 Department of Statistics and Operations Research, Faculty of Science, King Saud University, P.O. Box 2455, Riyadh, 11451 Saudi Arabia

**Keywords:** Multivalent functions, Convolution, Derivative operators, Materials science, Mathematics and computing

## Abstract

In this paper, the new subclass $\mathcal {S}^n_{b,\lambda ,\delta ,p} ({\alpha })$ of a linear differential operator’s $\mathcal {N}_{\lambda ,\delta ,p}^{n}f(\zeta )$ associated with multivalent analytical function has been introduced. Further, the coefficient inequalities, extreme points for the extremal function, sharpness of the growth and distortion bounds, partial sums, starlikeness, and convexity of the subclass is investigated.

## Introduction

Assume that $\mathcal {U}:\left| {\zeta }\right| <1$ is the unit circle and that $f(\zeta )$ is an analytical function, as exhibited by the power series.

1 $$\begin{aligned} w=f(\zeta )=\sum _{\nu =0}^{\infty }b_n{\zeta }^n=b_0+b_1\zeta +b_2{\zeta }^2+\cdots , \end{aligned}$$

The sequence $\lbrace b_n \rbrace$ of coefficients in (1) is the basis for the function $f(\zeta )$, which maps $\mathcal {U}$ onto a sub-domain $\mathcal {S}$ of a Riemann surface.

An attribute of geometry of $\mathcal {S}$ is described by the statement that the univalent function $f(\zeta )$ is in $\mathcal {U}$. By definition, $f(\zeta )$ has the property of being univalent in $\mathcal {U}$ if

2 $$\begin{aligned} f(\zeta _1) = f(\zeta _2),\ \ \ \zeta _1,\zeta _2 \in \mathcal {U} \implies \zeta _1=\zeta _2. \end{aligned}$$

Briefly, $f(\zeta )$ is said to be univalent in $\mathcal {U}$ if it does not take any value more than once for $\zeta$ in $\mathcal {U}$.

The image of $\mathcal {U}$ creates a simple domain in the *w*-plane, provided $f(\zeta )$ is univalent.

The multivalent function is a logical consequence of the idea of the univalent function. Assume that $p\in \mathcal {N}$. It is said that $f(\zeta )=w_0$ has *p* roots in $\mathcal {U}$ and that the function $f(\zeta )$ denotes *p*-valent in $\mathcal {U}.$ Meanwhile, the constraints

3 $$\begin{aligned} f(\zeta _1) = f(\zeta _2)=\cdots ,= f(\zeta _{p+1}),\ \ \ \zeta _1,\zeta _2, \ldots \zeta _{p+1} \in \mathcal {U} \implies \zeta _i\ne \zeta _j. \end{aligned}$$

for a certain pair, ensure that $i\ne j$. To put it simply, $f(\zeta )$ is *p*-valent in $\mathcal {U}$ assuming that some value but no value exceeds *p* times.

Typically, 1907 the work of Koebe^20^ was considered the earliest stages of the concept of univalent functions. In 1933 by Montel^21^ and in 1938 by Biernacki^7^ were given two credible evaluations of the research on univalent and multivalent functions. After that, the volume of information grew rapidly, as usual, making it challenging for researchers to ascertain the current situation. Books from Schaeffer and Spencer^26^, Jenkins^19^, and others explore specialized parts of the topic in great detail. The writings by Hayman^18^ and Goluzin^15^ provided a thorough overview, and it contained enough unresolved issues for a while. The study of fragments by Bernardi^6^ and Hayman^17^ offered additional direction in the field.

The differential and integral operators of normalized analytic functions have recently gained a lot of popularity. Numerous articles covered the operators and generalizations made by various authors. In 1975, Ruscheweyh^24^ introduced the differential operator and it is generalized by Salagean^25^ in 1985. For a long time, these two operators were utilized to investigate various subclasses of univalent function by researchers. In the year of 2004 Al-Oboudi’s^2^ generalized of the Salagean operator, followed by Shaqsi and Darus^3,4^ generalized the Ruscheweyh and Salagean differential operators in 2008. Following that, several authors began to develop new operators based on the Salagean and Ruscheweyh in their own distinctive style. For example, see^5,8–10,12,13,16,22,23,27–31^. By the help of this survey, in this current work, certain properties of subclass of new linear differential operator of multivalent functions have been investigated.

Let $\mathcal {A}_p$ be called a class of multivalent analytic functions

4 $$\begin{aligned} {f}({\zeta })={\zeta }^p+\sum ^{\infty }_{\nu =p+1} a_\nu {\zeta }^\nu , \end{aligned}$$

belongs to $$\mathcal {U} =\lbrace \zeta :|\zeta |<1\rbrace .$$

For ${f}(\zeta )\in \mathcal {A}_p$, Aghalary et al.^1^ studied the following multiplier transformation operator

5 $$\begin{aligned} \mathcal {I}_{p}(n,\lambda )= \zeta ^p+\sum ^{\infty }_{\nu =p+1}\left( \frac{\nu +\lambda }{p+\lambda }\right) ^n a_{\nu }\zeta ^{\nu }, \ \ (\lambda \ge 0 ). \end{aligned}$$

For ${f}(\zeta )\in \mathcal {A}_p,$ a new differential operator has defined $\mathcal {N}_{\lambda ,\delta ,p}^{n}(f(\zeta )) = \mathcal {I}_{p}(n,\lambda )*f (\zeta )$ by

$$\mathcal {N}_{\lambda ,\delta ,p}^{0}=\zeta ^p+\sum ^{\infty }_{\nu =p+1} a_{\nu }\zeta ^{\nu }$$

$$\mathcal {N}_{\lambda ,\delta ,p}^{1}=\left( 1-\delta \right) \mathcal {I}_{p}(1,\lambda )+\frac{\delta \zeta }{p} \left( \mathcal {I}_{p}(1,\lambda ) \right) '= \zeta ^p+\sum ^{\infty }_{\nu =p+1}\left[ \frac{p(\lambda +\nu )+\left( \nu -p\right) (\nu +\lambda )\delta }{p(p+\lambda )}\right] a_{\nu } \zeta ^{\nu }$$

$$ \mathcal {N}_{\lambda ,\delta ,p}^{2}=\mathcal {N}_{\lambda ,\delta ,p}\left( \mathcal {N}_{\lambda ,\delta ,p}^{1}\right) $$ Similarly,

6 $$\begin{aligned} \mathcal {N}_{\lambda ,\delta ,p}^{n}=\mathcal {N}_{\lambda ,\delta ,p}\left( \mathcal {N}_{\lambda ,\delta ,p}^{n-1}\right) =\zeta ^p+\sum ^{\infty }_{\nu =p+1}\left[ \frac{p(\lambda +\nu )+(\nu -p)(\lambda +\nu )\delta }{p(p+\lambda )}\right] ^n a_{\nu } \zeta ^{\nu },( \lambda , \delta \ge 0, n \in N_0). \end{aligned}$$

### Remark 1.1

For $\delta =0$ in (6), the multiplier transformations $I_{p}(n,\lambda )$ are obtained. It was stated by Aghalary et al.^1^.

For $\delta =0, p=1$ in (6), the operator $\mathcal {I}^{n}_\lambda$ is obtained. It was presented by Cho and Srivastava^11^.

For $\delta =0, p=1, \lambda =1$ in (6), the differential oprator $\mathcal {I}^{n}$ was introduced by Uralegaddi et al.^32^.

The operator $\mathcal {D}^{n}$ is stated by Salagean^25^ for $\lambda =0, \delta =0, p=1$ in (6).

For $\lambda =0, \delta =0, p=1, n=-n$ in (6), the multiplier transformation $I^{-n}$ is obtained; it was introduced by Flett^14^.

## The class $\mathcal {S}^n_{b,\lambda ,\delta ,p } ({\alpha })$

### Definition 2.1

Let $\mathcal {S}^{n}_{b,\lambda ,\delta ,p } (\phi (\zeta ))$ denote the subclass of ${f}(\zeta )\in {\mathcal {A}_p},$ in which

7 $$\begin{aligned} 1+\frac{1}{b}\left( \frac{\frac{\zeta }{p}(\mathcal {N}_{\lambda ,\delta ,p}^{n})'}{\mathcal {N}^{n}_{\lambda ,\delta ,p} }-1\right) \prec \phi (\zeta ). \end{aligned}$$

### Definition 2.2

Let $\mathcal { S}^{n}_{b,\lambda ,\delta ,p } (\phi (\zeta )) \equiv \mathcal {S}^{n}_{b,\lambda ,\delta ,p } ({\alpha })$ represents a subclass belonging to

$${f}(\zeta )\in {\mathcal {A}_p},$$ then

8 $$\begin{aligned} Re\left( 1+\frac{1}{b}\left( \frac{\frac{\zeta }{p}(\mathcal {N}_{\lambda ,\delta ,p}^{n})'}{\mathcal {N}^{n}_{\lambda ,\delta ,p} }-1\right) \right) >{\alpha }. \end{aligned}$$

where $\phi (\zeta )=\frac{1+(1-2{\alpha })\zeta }{(1-\zeta )}$, $n\in {N_0}, 0\le \alpha <1, \lambda ,\delta \ge 0,b \in C-\lbrace 0 \rbrace$ and all $\zeta \in {\mathcal {U}}.$

### Estimate the coefficient inequality

The concepts of univalent and multivalent functions are crucial while studying complex analysis. They are usually defined on the complex plane. It is customary in this context to estimate the coefficients of these functions, more precisely, their inequalities. We will gain insight into the branching structure of multivalent functions by estimating their coefficients. The coefficient inequalities provide information about how branch points behave over the complex plane of the function. In both cases, understanding the coefficients and their inequalities in univalent and multivalent functions are essential for various applications in complex analysis, particularly in the fields of conformal mapping, complex geometry, and Riemann surfaces. The coefficient estimation provides valuable information about the behavior of functions and its geometric properties, helping mathematicians and scientists work with them effectively in various contexts.

#### Theorem 2.1

Let $${f}(\zeta )\in \mathcal {S}^{n}_{b,\lambda ,\delta ,p } ({\alpha })$$ , then

9 $$\begin{aligned} \sum ^{\infty }_{\nu =p+1} \left| \frac{\alpha bp-\nu +p-pb}{p}\right| \left[ \frac{p(\lambda +\nu )+(\nu -p)(\lambda +\nu )\delta }{p(p+\lambda )}\right] ^n \left| a_{\nu }\right| \le (1-{\alpha })\left| b\right| . \end{aligned}$$

#### Proof

Let

$$\begin{aligned} F(\zeta ){} & {} =1+\frac{1}{b}\left( \frac{\frac{\zeta }{p}(\mathcal {N}_{\lambda ,\delta ,p}^{n})'}{\mathcal {N}_{\lambda ,\delta ,p}^{n} }-1\right) -\alpha \\{} & {} =1+ \frac{\frac{\zeta }{p}(\mathcal {N}_{\lambda ,\delta ,p}^{n})'-(1+\alpha b)\mathcal {N}_{\lambda ,\delta ,p}^{n}}{b \mathcal {N}_{\lambda ,\delta ,p}^{n} } \end{aligned}$$

By the condition of the class,

$$\begin{aligned} F(\zeta )\prec \frac{1+\zeta }{1-\zeta }. \end{aligned}$$

There exist a schwarz function $w(\zeta )$, with $w(0)=0$ and $\left| w \right| < 1,$ such that

$$\begin{aligned} F(\zeta )= \frac{1+w(\zeta )}{1-w(\zeta )}. \end{aligned}$$

This implies that

$$\begin{aligned} w(\zeta )=\frac{F(\zeta )-1}{F(\zeta )+1}. \end{aligned}$$

We know that

$$\begin{aligned} \left| w(\zeta )\right| =\left| \frac{F(\zeta )-1}{F(\zeta )+1} \right| < 1. \end{aligned}$$

Then

$$\begin{aligned} \left| \frac{F(\zeta )-1}{F(\zeta )+1} \right|{} & {} =\left| \frac{\frac{\zeta }{p}(\mathcal {N}_{\lambda ,\delta ,p}^{n})'-(1+\alpha b)\mathcal {N}_{\lambda ,\delta ,p}^{n}}{\frac{\zeta }{p}(\mathcal {N}_{\lambda ,\delta ,p}^{n})'-(1+\alpha b-2b)\mathcal {N}_{\lambda ,\delta ,p}^{n} } \right| \\{} & {} =\left| \frac{\zeta ^p+\sum _{\nu =p+1}^{\infty }\frac{\nu }{p} c_\nu a_\nu \zeta ^\nu -(1+\alpha b)\zeta ^p-\sum _{\nu =p+1}^{\infty }(1+\alpha b) c_\nu a_\nu \zeta ^\nu }{\zeta ^p+\sum _{\nu =p+1}^{\infty }\frac{\nu }{p} c_\nu a_\nu \zeta ^\nu -(1+\alpha b-2b)\zeta ^p-\sum _{\nu =p+1}^{\infty }(1+\alpha b-2b) c_\nu a_\nu \zeta ^\nu } \right| \\{} & {} =\left| \frac{-\alpha b -\sum _{\nu =p+1}^{\infty }(1+\alpha b-\frac{\nu }{p}) c_\nu a_\nu \zeta ^{\nu -p}}{(2-\alpha )b-\sum _{\nu =p+1}^{\infty }(1+\alpha b-2b-\frac{\nu }{p}) c_\nu a_\nu \zeta ^{\nu -p} } \right| \\{} & {} \le \frac{\alpha \left| b \right| +\sum _{\nu =p+1}^{\infty }\left| (1+\alpha b-\frac{\nu }{p})\right| c_\nu \left| a_\nu \right| \left| \zeta ^{\nu -p}\right| }{(2-\alpha )\left| b\right| -\sum _{\nu =p+1}^{\infty }\left| (1+\alpha b-2b-\frac{\nu }{p})\right| c_\nu \left| a_\nu \right| \left| \zeta ^{\nu -p}\right| }. \end{aligned}$$

The last expression is bounded by 1, if

$$\begin{aligned} \alpha \left| b \right| +\sum _{\nu =p+1}^{\infty }\left| (1+\alpha b-\frac{\nu }{p})\right| c_\nu \left| a_\nu \right| \le (2-\alpha )\left| b \right| -\sum _{\nu =p+1}^{\infty }\left| (1+\alpha b-2b-\frac{\nu }{p})\right| c_\nu \left| a_\nu \right| . \end{aligned}$$

Which implies that,

$$\begin{aligned} \sum _{\nu =p+1}^{\infty }\left| \left( 1+\alpha b-b-\frac{\nu }{p}\right) \right| c_\nu \left| a_\nu \right| \le (1-\alpha )\left| b \right| , \end{aligned}$$

where Hence the equation (9) is hold.$\square$

#### Corollary 2.1

Let $${f}\in \mathcal {S}^n_{b,\lambda ,\delta ,p } ({\alpha }),$$ then

10 $$\begin{aligned} \left| a_{\nu }\right| \le \frac{(1-{\alpha })\left| b \right| }{\left| \frac{\alpha bp-\nu +p-pb}{p}\right| \left[ \frac{p(\lambda +\nu )+(\nu -p)(\lambda +\nu )\delta }{p(p+\lambda )}\right] ^n}, \end{aligned}$$

and the equality is concluded for the function ${f}(\zeta )$ is given by

11 $$\begin{aligned} {f}(\zeta )=\zeta ^p+\frac{(1-{\alpha })\left| b \right| }{\left| \frac{\alpha bp-\nu +p-pb}{p}\right| \left[ \frac{p(\lambda +\nu )+(\nu -p)(\lambda +\nu )\delta }{p(p+\lambda )}\right] ^n}\zeta ^{\nu }, \ \ \ \nu \ge p+1. \end{aligned}$$

### Extreme points

Extremal points are analyses in the framework of multivalent functions in order to comprehend branch cuts, singularities, and branching behavior. It is essential to comprehending the function of complex structure and Riemann surface.

#### Theorem 2.2

Let

$$\begin{aligned} {f}_p({\zeta })={\zeta }^p, {f}_\nu ({\zeta })={\zeta }^p+\eta _\nu \frac{(1-{\alpha })\left| b \right| }{C(\lambda )}{\zeta }^\nu , \nu =p+1,p+2,\ldots , \end{aligned}$$

where

$$\begin{aligned} C(\lambda )=\sum ^{\infty }_{\nu =p+1} \left| \frac{\alpha bp-\nu +p-pb}{p}\right| \left[ \frac{p(\lambda +\nu )+(\nu -p)(\lambda +\nu )\delta }{p(p+\lambda )}\right] ^n. \end{aligned}$$

Then ${f}\in \mathcal {S}^n_{b,\lambda ,\delta ,p } ({\alpha })$ only when it is in the form

$$\begin{aligned} {f}({\zeta })=\eta _p {f}_p({\zeta }) + \sum _{\nu =p+1}^{\infty } \eta _\nu {f}_\nu ({\zeta }), \end{aligned}$$

where $$\eta _\nu \ge 0$$ and $$\eta _p=1-\sum _{\nu =p+1}^{\infty }\eta _\nu .$$

#### Proof

Let assume that

$$\begin{aligned} {f}({\zeta })=\eta _p {f}_p({\zeta }) + \sum _{\nu =p+1}^{\infty }\eta _\nu {f}_\nu ({\zeta }). \end{aligned}$$

Then

$$\begin{aligned} {f}({\zeta }){} & {} =\left( 1-\sum _{\nu =p+1}^{\infty }\eta _\nu \right) {\zeta }^p+\sum _{\nu =p+1}^{\infty } \eta _\nu \left( {\zeta }^p+\frac{(1-{\alpha })\left| b \right| }{C(\lambda )}{\zeta }^\nu \right) \\{} & {} ={\zeta }^p+\sum ^{\infty }_{\nu =p+1}\eta _\nu \frac{(1-{\alpha })\left| b \right| }{C(\lambda )}{\zeta }^\nu \\{} & {} ={\zeta }^p+\sum ^{\infty }_{\nu =p+1}a_\nu {\zeta }^\nu \end{aligned}$$

Thus,

$$\begin{aligned}{} & {} \sum ^{\infty }_{\nu =p+1}{C(\lambda )}\left| a_{\nu }\right| \\{} & {} =\sum ^{\infty }_{\nu =p+1}C(\lambda )\eta _\nu \frac{(1-{\alpha })\left| b \right| }{C(\lambda )} \\{} & {} =(1-{\alpha })\left| b \right| \sum _{\nu =p+1}^{\infty }\eta _\nu \\{} & {} =(1-{\alpha })\left| b \right| (1-\eta _p) \\{} & {} \quad <(1-{\alpha })\left| b \right| , \end{aligned}$$

which demonstrates

$$\begin{aligned} {f}\in \mathcal {S}^n_{b,\lambda ,\delta ,p } ({\alpha }). \end{aligned}$$

Conversely,

Consider this

$$\begin{aligned} {f}\in \mathcal {S}^n_{b,\lambda ,\delta ,p } ({\alpha }). \end{aligned}$$

While

$$\begin{aligned} \left| a_\nu \right| \le \frac{(1-{\alpha })\left| b \right| }{C(\lambda )},\nu =p+1,p+2,\ldots \end{aligned}$$

Let

$$\begin{aligned} \eta _\nu \le \frac{C(\lambda )}{(1-{\alpha })\left| b \right| }a_\nu ,\eta _p=1-\sum ^{\infty }_{\nu =p+1}\eta _\nu . \end{aligned}$$

Thus,

$$\begin{aligned} {f}({\zeta }){} & {} =\zeta ^p+\sum ^{\infty }_{\nu =p+1}a_\nu \zeta ^\nu \\ {f}({\zeta }){} & {} =(\eta _p+\sum ^{\infty }_{\nu =p+1}\eta _\nu )\zeta ^p+\sum ^{\infty }_{\nu =p+1}\eta _\nu \frac{(1-{\alpha })\left| b \right| }{C(\lambda )} \zeta ^\nu \\{} & {} = \eta _p {f}_p({\zeta }) + \sum _{\nu =p+1}^{\infty }\eta _\nu \lbrace {\zeta }^\nu +\frac{(1-{\alpha })\left| b \right| }{C(\lambda )}\zeta ^\nu \rbrace \\{} & {} = \eta _p {f}_p({\zeta }) + \sum _{\nu =p+1}^{\infty }\eta _\nu {f}_\nu ({\zeta }). \end{aligned}$$

$$\square $$

### Growth and distortion theorems

Growth and distortion theorems are useful tools in the study of univalent and multivalent functions because they help to characterize and comprehend the behavior of these functions and how they relate to the geometry of the complex plane. According to the growth theorem, a complex-valued function is inherently constant if it is entire and bounded. The geometry of curves and regions in the complex plane is influenced by analytic functions, as revealed by the distortion theorem. It sets limits on the maximum amount of stretching or distortion that can happen when a function transfers a region or curve from one domain to another. By using these theorems, mathematicians and researchers can study the behavior of complex analytic functions and how it impacts the sizes and shapes of curves and regions in the complex plane.

#### Theorem 2.3

If $$f\in \mathcal {S}^n_{b,\lambda ,\delta ,p } ({\alpha })$$,then

$$\begin{aligned} \rho ^p-\frac{(1-{\alpha })\left| b \right| }{\left| \frac{\alpha bp-1-bp}{p}\right| \left( \frac{(p+1+\lambda )(p+\delta )}{p(p+\lambda )}\right) ^n} \rho ^{p+1}\le \left| f(\zeta )\right| \le \rho ^p+\frac{(1-{\alpha })\left| b \right| }{\left| \frac{\alpha bp-1-bp}{p}\right| \left( \frac{(p+1+\lambda )(p+\delta )}{p(p+\lambda )}\right) ^n} \rho ^{p+1}, \end{aligned}$$

$\left| {\zeta }\right| =\rho <1,$ provided $\nu \ge p+1.$ The result called as sharp for

$$\begin{aligned} f(\zeta )=\zeta ^p+\frac{(1-{\alpha })\left| b \right| }{\left| \frac{\alpha bp-1-bp}{p}\right| \left( \frac{(p+1+\lambda )(p+\delta )}{p(p+\lambda )}\right) ^n}\zeta ^{p+1}. \end{aligned}$$

#### Proof

By making use of the inequality (9) for $f\in \mathcal {S}^n_{b,\lambda ,\delta ,p } ({\alpha })$ together with

$$\begin{aligned} \left| \frac{\alpha bp-1-bp}{p}\right| \left( \frac{(p+1+\lambda )(p+\delta )}{p(p+\lambda )}\right) ^n \le \left| \frac{\alpha bp-\nu +p-pb}{p}\right| \left( \frac{p(\lambda +\nu )+(\nu -p)(\lambda +\nu )\delta }{p(p+\lambda )}\right) ^n, \end{aligned}$$

then

$$\begin{aligned}{} & {} {\left| \frac{\alpha bp-1-bp}{p}\right| \left( \frac{(p+1+\lambda )(p+\delta )}{p(p+\lambda )}\right) ^n}\sum _{\nu =p+1}^{\infty }a_\nu \\{} & {} \le {\left| \frac{\alpha bp-\nu +p-pb}{p}\right| \left( \frac{p(\lambda +\nu )+(\nu -p)(\lambda +\nu )\delta }{p(p+\lambda )}\right) ^n} \sum _{\nu =p+1}^{\infty }a_\nu \le (1-{\alpha })\left| b \right| . \end{aligned}$$

12 $$\begin{aligned} \sum _{\nu =p+1}^{\infty }a_\nu \le \frac{(1-{\alpha })\left| b \right| }{\left| \frac{\alpha bp-1-bp}{p}\right| \left( \frac{(p+1+\lambda )(p+\delta )}{p(p+\lambda )}\right) ^n}. \end{aligned}$$

By using (12) for the function $f(\zeta )=\zeta ^p+\sum _{\nu =p+1}^{\infty }a_\nu \zeta ^\nu \in \mathcal {S}^n_{b,\lambda ,\delta ,p } ({\alpha })$, since $|\zeta |=\rho ,$

$$\begin{aligned} |f(\zeta )|{} & {} =\rho ^p+\sum _{\nu =p+1}^{\infty }a_\nu \rho ^\nu \\{} & {} \le \rho ^p+\rho ^{p+1}\sum _{\nu =p+1}^{\infty }a_\nu \\{} & {} \le \rho ^p+\frac{(1-{\alpha })\left| b \right| }{\left| \frac{\alpha bp-1-bp}{p}\right| \left( \frac{(p+1+\lambda )(p+\delta )}{p(p+\lambda )}\right) ^n}\rho ^{p+1}, \end{aligned}$$

and similarly,

$$\begin{aligned} |f(\zeta )| \ge \rho ^p-\frac{(1-{\alpha })\left| b \right| }{\left| \frac{\alpha bp-1-bp}{p}\right| \left( \frac{(p+1+\lambda )(p+\delta )}{p(p+\lambda )}\right) ^n}\rho ^{p+1}. \end{aligned}$$

$$\square $$

#### Theorem 2.4

If $$f\in \mathcal {S}^n_{b,\lambda ,\delta ,p } ({\alpha })$$,then

$$\begin{aligned} p\rho ^{p-1}-\frac{(p+1)(1-{\alpha })\left| b \right| }{\left| \frac{\alpha bp-1-bp}{p}\right| \left( \frac{(p+1+\lambda )(p+\delta )}{p(p+\lambda )}\right) ^n} \rho ^{p}\le \left| f'(\zeta )\right| \le p\rho ^{p-1}+\frac{(p+1)(1-{\alpha })\left| b \right| }{\left| \frac{\alpha bp-1-bp}{p}\right| \left( \frac{(p+1+\lambda )(p+\delta )}{p(p+\lambda )}\right) ^n} \rho ^{p}, \end{aligned}$$

$\left| {\zeta }\right| =\rho <1,$ provided $\nu \ge p+1.$ Clearly, the outcome is sharp for

$$\begin{aligned} f(\zeta )=\zeta ^p+\frac{(1-{\alpha })\left| b \right| }{\left| \frac{\alpha bp-1-bp}{p}\right| \left( \frac{(p+1+\lambda )(p+\delta )}{p(p+\lambda )}\right) ^n}\zeta ^{p+1}. \end{aligned}$$

#### Proof

By using the inequality (9) for $f\in \mathcal {S}^n_{b,\lambda ,\delta ,p } ({\alpha })$, then

$$\begin{aligned} \sum _{\nu =p+1}^{\infty }a_\nu \le \frac{(1-{\alpha })\left| b \right| }{\left| \frac{\alpha bp-1-bp}{p}\right| \left( \frac{(p+1+\lambda )(p+\delta )}{p(p+\lambda )}\right) ^n}. \end{aligned}$$

By using (12), then

$$\begin{aligned} \sum _{\nu =p+1}^{\infty }\nu a_\nu \le \frac{(p+1)(1-{\alpha })\left| b \right| }{\left| \frac{\alpha bp-1-bp}{p}\right| \left( \frac{(p+1+\lambda )(p+\delta )}{p(p+\lambda )}\right) ^n}. \end{aligned}$$

For the function $f(\zeta )=\zeta ^p+\sum _{\nu =p+1}^{\infty }a_\nu \zeta ^\nu \in \mathcal {S}^n_{b,\lambda ,\delta ,p } ({\alpha })$, then

$$\begin{aligned} |f'(\zeta )|{} & {} =p\rho ^{p-1}+\sum _{\nu =p+1}^{\infty }\nu a_\nu \rho ^{\nu -1} \\{} & {} \le p\rho ^{p-1}+\rho ^{p}\sum _{\nu =p+1}^{\infty }\nu a_\nu \\{} & {} \le p\rho ^{p-1}+\frac{(p+1)(1-{\alpha })\left| b \right| }{\left| \frac{\alpha bp-1-bp}{p}\right| \left( \frac{(p+1+\lambda )(p+\delta )}{p(p+\lambda )}\right) ^n}\rho ^{p}, \end{aligned}$$

and similarly,

$$\begin{aligned} |f'(\zeta )| \ge p\rho ^{p-1}-\frac{(p+1)(1-{\alpha })\left| b \right| }{\left| \frac{\alpha bp-1-bp}{p}\right| \left( \frac{(p+1+\lambda )(p+\delta )}{p(p+\lambda )}\right) ^n}\rho ^{p}. \end{aligned}$$

$$\square $$

### Convexity and starlikeness

The coefficient inequalities of power series functions are frequently caused by starlikeness and convexity. Starlike functions fulfill the well-known Bieberbach conjecture, which gives restriction on the coefficients of starlike function. The geometric shapes can be preserved by mapping functions that are starlike or convex. The starlikeness and convexity of multivalent functions maintain specific structures, these qualities are crucial.

#### Theorem 2.5

Let $f\in \mathcal {S}^n_{b,\lambda ,\delta ,p } ({\alpha }),$ then the subclass claimed as convex .

#### Proof

Let

$$\begin{aligned} {f}_j({\zeta })={\zeta }^p+\sum _{\nu =p+1}^{\infty }a_{\nu ,j}{\zeta }^\nu ,a_{\nu ,j}\ge 0,j=1,2, \end{aligned}$$

contains $${f}\in \mathcal {S}^n_{b,\lambda ,\delta ,p } ({\alpha }).$$

it is necessary to show that

$$\begin{aligned} h({\zeta })=(\tau +1){f}_1({\zeta })-\tau {f}_2({\zeta }),0\le {\tau } \le 1. \end{aligned}$$

while

$$\begin{aligned} h({\zeta })={\zeta }^p+\sum _{\nu =p+1}^{\infty }\left[ (1+\tau )a_{\nu ,1}-\tau a_{\nu ,2}\right] {\zeta }^\nu , \end{aligned}$$

which implies that

$$\begin{aligned} \sum _{\nu =p+1}^{\infty }{} & {} \left| \frac{\alpha bp-\nu +p-pb}{p}\right| \left[ \frac{p(\lambda +\nu )+(\nu -p)(\lambda +\nu )\delta }{p(p+\lambda )}\right] ^n(1+\tau )a_{\nu ,1} \\{} & {} \quad -\left| \frac{\alpha bp-\nu +p-pb}{p}\right| \left[ \frac{p(\lambda +\nu )+(\nu -p)(\lambda +\nu )\delta }{p(p+\lambda )}\right] ^n\tau a_{\nu ,2} \\{} & {} \le (1+\tau )(1-{\alpha })\left| b \right| -\tau (1-{\alpha })\left| b \right| \end{aligned}$$

$$\begin{aligned} \le (1-{\alpha })\left| b \right| , \end{aligned}$$

Thus

$$\begin{aligned} h\in \mathcal {S}^n_{b,\lambda ,\delta ,p } ({\alpha }). \end{aligned}$$

Hence $\mathcal {S}^n_{b,\lambda ,\delta ,p } ({\alpha })$ called convex. $\square$

#### Theorem 2.6

If ${f}\in \mathcal {S}^n_{b,\lambda ,\delta ,p } ({\alpha })$ , then according to order ${\varsigma }$ *f* is *p*-valently convex in the disc $\left| {\zeta }\right| <\rho _2$, where

$$\begin{aligned} \rho _2:=inf\left( \frac{p({\varsigma -p})\left( \frac{p b{\alpha }-\nu +p-p b}{p}\right) \left[ \frac{p(\lambda +\nu )+(\nu -p)(\lambda +\nu )\delta }{p(p+\lambda )}\right] ^n}{\nu (\nu -{\varsigma })(1-{\alpha })\left| b \right| }\right) ^{\frac{1}{\nu -p}}, (\nu \ge p+1). \end{aligned}$$

The bound for $\left| {\zeta }\right|$ is sharp for each $\nu$,with the form (11) serving as the extreme function.

#### Proof

If ${f}\in \mathcal {S}^n_{b,\lambda ,\delta ,p } ({\alpha }),$ and *f* is claimed orderly convex of ${\varsigma },$ then it is required to prove that

13 $$\begin{aligned} \left| 1+\frac{\zeta f''(\zeta )}{f'(\zeta )}-p\right|<p-{\varsigma } \ \ for \left| {\zeta }\right| <\rho _2. \end{aligned}$$

Now, the equation (13) gives

14 $$\begin{aligned} \left| 1+\frac{\zeta f''(\zeta )}{f'(\zeta )}-p\right| =\left| \frac{f'(\zeta )+\zeta f''(\zeta )-pf'(\zeta )}{f'(\zeta )}\right| \le \frac{\sum ^{\infty }_{\nu =p+1}\nu (\nu -p)a_\nu \left| \zeta \right| ^{\nu -p}}{p+\sum ^{\infty }_{\nu =p+1}\nu a_\nu \left| \zeta \right| ^{\nu -p}}. \end{aligned}$$

From (13) and (14), derive

15 $$\begin{aligned} \sum ^{\infty }_{\nu =p+1}\frac{\nu (\nu -{\varsigma })}{p({\varsigma }-p)}a_\nu \left| \zeta \right| ^{\nu -p}\le 1. \end{aligned}$$

In the view of (13), it follows that (15) is true if

16 $$\begin{aligned} \left| {\zeta }\right| \le \left( \frac{p({\varsigma }-p)\left| \frac{\alpha bp-\nu +p-pb}{p}\right| \left[ \frac{p(\lambda +\nu )+(\nu -p)(\lambda +\nu )\delta }{p(p+\lambda )}\right] ^n}{\nu (\nu -{\varsigma })(1-{\alpha })\left| b \right| }\right) ^{\frac{1}{\nu -p}}, (\nu \ge p+1). \end{aligned}$$

Setting $\left| {\zeta }\right| =\rho _2$ in (16), the result follows. The sharpness can be verified. $\square$

#### Theorem 2.7

If ${f}\in \mathcal {S}^n_{b,\lambda ,\delta ,p } ({\alpha })$ , then according to order ${\varsigma },$*f* is *p*-valently starlike $(0\le {\varsigma }<p)$ in the disc $\left| {\zeta }\right| <\rho _3$, where

$$\begin{aligned} \rho _3:=inf\left( \frac{({\varsigma }-p)\left( \frac{p b{\alpha }-\nu +p-p b}{p}\right) \left[ \frac{p(\lambda +\nu )+(\nu -p)(\lambda +\nu )\delta }{p(p+\lambda )}\right] ^n}{(\nu -{\varsigma })(1-{\alpha })\left| b \right| }\right) ^{\frac{1}{\nu -p}}, (\nu \ge p+1). \end{aligned}$$

The bound for $\left| {\zeta }\right|$ is sharp for each $\nu$, with the form (11) serving as the extreme function.

#### Proof

If ${f}\in \mathcal {S}^n_{b,\lambda ,\delta ,p } ({\alpha }),$ and *f* is claimed orderly starlike of ${\varsigma },$ then it is required to demonstrate that

17 $$\begin{aligned} \left| \frac{\zeta f'(\zeta )}{f(\zeta )}-p\right|<p-{\varsigma } \ \ for \left| {\zeta }\right| <\rho _3. \end{aligned}$$

Now, the equation (17) gives

18 $$\begin{aligned} \left| \frac{\zeta f'(\zeta )}{f(\zeta )}-p\right| =\left| \frac{\zeta f'(\zeta )-pf(\zeta )}{f(\zeta )}\right| \le \frac{\sum ^{\infty }_{\nu =p+1}(\nu -p)a_\nu \left| \zeta \right| ^{\nu -p}}{1+\sum ^{\infty }_{\nu =p+1}a_\nu \left| \zeta \right| ^{\nu -p}}. \end{aligned}$$

From (17) and (18), the following equation obtain

19 $$\begin{aligned} \sum ^{\infty }_{\nu =p+1}\frac{(\nu -{\varsigma })}{({\varsigma }-p)}a_\nu \left| \zeta \right| ^{\nu -p}\le 1. \end{aligned}$$

In the view of (17), it follows that (19) is true if

20 $$\begin{aligned} \left| {\zeta }\right| \le \left( \frac{({\varsigma }-p)\left| \frac{\alpha bp-\nu +p-pb}{p}\right| \left[ \frac{p(\lambda +\nu )+(\nu -p)(\lambda +\nu )\delta }{p(p+\lambda )}\right] ^n}{(\nu -{\varsigma })(1-{\alpha })\left| b \right| }\right) ^{\frac{1}{\nu -p}}, (\nu \ge p+1). \end{aligned}$$

Setting $\left| {\zeta }\right| =\rho _3$ in (20), the result follows. The sharpness can be verified. $\square$

### Partial sums

The concept of partial sums is one that is commonly used in the study of infinite series. On the other hand, partial sums are useful in complicated analysis and can be used in many other mathematical situations, including function analysis. This section looks into the relationship between form (4) and its series of partial sums.

$$\begin{aligned} f(\zeta )=\zeta ^p \end{aligned}$$

and

$$\begin{aligned} f_\nu (\zeta )=\zeta ^p+\sum ^{n}_{\nu =p+1}a_\nu \zeta ^\nu , \nu =p+1,p+2,p+3,\ldots , \end{aligned}$$

when the coefficients are small enough to satisfy the analogous condition

$$\begin{aligned} \sum ^{\infty }_{\nu =p+1} \left| \frac{\alpha bp-\nu +p-pb}{p}\right| \left[ \frac{p(\lambda +\nu )+(\nu -p)(\lambda +\nu )\delta }{p(p+\lambda )}\right] ^n \left| a_\nu \right| \le (1-{\alpha })\left| b \right| . \end{aligned}$$

It can be written as

$$\begin{aligned} \sum ^{\infty }_{\nu =p+1} \mathcal {X}_\nu \left| a_\nu \right| \le 1, \end{aligned}$$

where

$$\begin{aligned} \mathcal {X}_\nu =\frac{\left( \left| \frac{\alpha bp-\nu +p-pb}{p}\right| \left[ \frac{p(\lambda +\nu )+(\nu -p)(\lambda +\nu )\delta }{p(p+\lambda )}\right] ^n \right) }{(1-{\alpha })\left| b \right| }. \end{aligned}$$

Then $$f\in \mathcal {S}^n_{b,\lambda ,\delta ,p } ({\alpha })$$.

#### Theorem 2.8

If $f\in \mathcal {S}^n_{b,\lambda ,\delta ,p } ({\alpha })$, satisfying (7),then

$$\begin{aligned} Re \left( \frac{{f(\zeta )}}{f_\nu (\zeta )}\right) \ge 1-\frac{1}{\mathcal {X}_{n+1}}. \end{aligned}$$

#### Proof

Clearly $$\mathcal {X}_{\nu +1}>\mathcal {X}_\nu >1,\nu =p+1, p+2, p+3,\ldots $$,

Utilising (4), to get

$$\begin{aligned} \sum ^{n}_{\nu =p+1}\left| a_\nu \right| +\mathcal {X}_{n+1}\sum ^{\infty }_{\nu =n+1}\left| a_\nu \right| \le \sum ^{\infty }_{\nu =p+1}\mathcal {X}_{\nu }\left| a_\nu \right| \le 1. \end{aligned}$$

Let

$$\begin{aligned} {\Phi }_1\left( {\zeta }\right) = \mathcal {X}_{n+1}\left[ \frac{{f(\zeta )}}{{f}_\nu (\zeta )}-\left( 1-\frac{1}{\mathcal {X}_{n+1}}\right) \right] \end{aligned}$$

$$\begin{aligned} =1+\frac{\mathcal {X}_{n+1} \sum ^{\infty }_{\nu =n+1} a_\nu \zeta ^{\nu -1}}{1+ \sum ^{n}_{\nu =p+1} a_\nu \zeta ^{\nu -1}}. \end{aligned}$$

Through basic computations, there is

$$\begin{aligned} \left| \frac{{\Phi }_1\left( {\zeta }\right) -1}{{\Phi }_1\left( {\zeta }\right) +1}\right| \le \frac{\mathcal {X}_{n+1} \sum ^{\infty }_{\nu =n+1} \left| a_\nu \right| }{2+2 \sum ^{n}_{\nu =p+1} \left| a_\nu \right| +\mathcal {X}_{n+1} \sum ^{\infty }_{\nu =n+1} \left| a_\nu \right| }\le 1, \end{aligned}$$

which gives,

$$\begin{aligned} Re \left( \frac{{f(\zeta )}}{f_{\nu }(\zeta )}\right) \ge 1-\frac{1}{\mathcal {X}_{n+1}}. \end{aligned}$$

Hence $f(\zeta )=\zeta +\frac{\zeta ^{n+1}}{\mathcal {X}_{n+1}}\bigg )$ will give the sharp result. $\square$

#### Theorem 2.9

If $f\in \mathcal {S}^n_{b,\lambda ,\delta ,p } ({\alpha })$ and satisfies (7). Then

$$\begin{aligned} Re \left( \frac{f_\nu (\zeta )}{{f(\zeta )}}\right) \ge \frac{\mathcal {X}_{n+1}}{1+\mathcal {X}_{n+1}}. \end{aligned}$$

#### Proof

Clearly $$\mathcal {X}_{\nu +1}>\mathcal {X}_\nu >1,\nu =p+1, p+2, p+3,\ldots $$.

Let

$$\begin{aligned} {\Phi }_2\left( {\zeta }\right){} & {} = \left( 1+\mathcal {X}_{n+1}\right) \left[ \frac{{f_\nu (\zeta )}}{f(\zeta )}-\left( \frac{\mathcal {X}_{n+1}}{1+\mathcal {X}_{n+1}}\right) \right] \\{} & {} =1+\frac{\left( 1+\mathcal {X}_{n+1}\right) \sum ^{\infty }_{\nu =n+1} a_\nu \zeta ^{\nu -1}}{1+ \sum ^{n}_{\nu =p+1} a_\nu \zeta ^{\nu -1}}. \end{aligned}$$

Through basic computations, there is

$$\begin{aligned} \left| \frac{{\Phi }_2\left( {\zeta }\right) -1}{{\Phi }_2\left( {\zeta }\right) +1}\right| \le \frac{\left( 1+\mathcal {X}_{n+1}\right) \sum ^{\infty }_{\nu =n+1} \left| a_\nu \right| }{2+2 \sum ^{n}_{\nu =p+1} \left| a_\nu \right| +\left( 1+\mathcal {X}_{n+1}\right) \sum ^{\infty }_{\nu =n+1} \left| a_\nu \right| }\le 1. \end{aligned}$$

Hence, the result

$$\begin{aligned} Re \left( \frac{{f_\nu (\zeta )}}{f(\zeta )}\right) \ge \frac{\mathcal {X}_{n+1}}{1+\mathcal {X}_{n+1}} \end{aligned}$$

is sharp for all *n*. $\square$

#### Theorem 2.10

If $f\in \mathcal {S}^n_{b,\lambda ,\delta ,p } ({\alpha })$, satisfying (7), then

$$\begin{aligned} Re \left( \frac{{f'(\zeta )}}{f{'_\nu }(\zeta )}\right) \ge 1-\frac{n+1}{\mathcal {X}_{n+1}}. \end{aligned}$$

#### Proof

Clearly $$\mathcal {X}_{\nu +1}>\mathcal {X}_\nu >1,\nu =p+1, p+2, p+3, \ldots $$.

Let

$$\begin{aligned} {\Phi }_3\left( {\zeta }\right){} & {} = \mathcal {X}_{n+1}\left[ \frac{{f'(\zeta )}}{{f'_\nu }(\zeta )}-\left( 1-\frac{n+1}{\mathcal {X}_{n+1}}\right) \right] \\{} & {} =1+\frac{\frac{\mathcal {X}_{n+1}}{n+1} \sum ^{\infty }_{\nu =n+1} \nu a_\nu \zeta ^{\nu -1}}{1+ \sum ^{n}_{\nu =p+1} \nu a_\nu \zeta ^{\nu -1}}. \end{aligned}$$

Through basic computations, there is

$$\begin{aligned} \left| \frac{{\Phi }_3\left( {\zeta }\right) -1}{{\Phi }_3\left( {\zeta }\right) +1}\right| \le \frac{\frac{\mathcal {X}_{n+1}}{n+1} \sum ^{\infty }_{\nu =n+1} \nu \left| a_\nu \right| }{2+2 \sum ^{n}_{\nu =p+1} \nu \left| a_\nu \right| +\frac{\mathcal {X}_{n+1}}{n+1} \sum ^{\infty }_{\nu =n+1} \nu \left| a_\nu \right| }\le 1, \end{aligned}$$

which gives,

$$\begin{aligned} Re \left( \frac{{f'(\zeta )}}{f{'_\nu }(\zeta )}\right) \ge 1-\frac{n+1}{\mathcal {X}_{n+1}}. \end{aligned}$$

Hence the result is sharp. $\square$

#### Theorem 2.11

If $f\in \mathcal {S}^n_{b,\lambda ,\delta ,p } ({\alpha })$, satisfying (7), then

$$\begin{aligned} Re \left( \frac{{f{'_\nu }(\zeta )}}{{f'}(\zeta )}\right) \ge \frac{\mathcal {X}_{n+1}}{n+1+\mathcal {X}_{n+1}}. \end{aligned}$$

#### Proof

Clearly $$\mathcal {X}_{\nu +1}>\mathcal {X}_\nu >1,\nu =p+1, p+2, p+3,\ldots $$.

Let

$$\begin{aligned} {\Phi }_4\left( {\zeta }\right){} & {} = \left( \left( n+1\right) +\mathcal {X}_{\nu }\right) \left[ \frac{{f'_\nu (\zeta )}}{{f'}(\zeta )}-\left( \frac{\mathcal {X}_{n+1}}{n+1+\mathcal {X}_{n+1}}\right) \right] \\{} & {} =1+\frac{\left( 1+\frac{\mathcal {X}_{n+1}}{n+1}\right) \sum ^{\infty }_{\nu =n+1}\nu a_\nu \zeta ^{\nu -1}}{1+ \sum ^{n}_{\nu =p+1}\nu a_\nu \zeta ^{\nu -1}}. \end{aligned}$$

Through basic computations, there is

$$\begin{aligned} \left| \frac{{\Phi }_4\left( {\zeta }\right) -1}{{\Phi }_4\left( {\zeta }\right) +1}\right| \le \frac{\left( 1+\frac{\mathcal {X}_{n+1}}{n+1}\right) \sum ^{\infty }_{\nu =n+1}\nu \left| a_\nu \right| }{2+2 \sum ^{n}_{\nu =p+1} \nu \left| a_\nu \right| +\left( 1+\frac{\mathcal {X}_{n+1}}{n+1}\right) \sum ^{\infty }_{\nu =n+1}\nu \left| a_\nu \right| }\le 1, \end{aligned}$$

which gives,

$$\begin{aligned} Re \left( \frac{{f{'_\nu }(\zeta )}}{{f'}(\zeta )}\right) \ge \frac{\mathcal {X}_{n+1}}{n+1+\mathcal {X}_{n+1}}. \end{aligned}$$

Hence the result is sharp. $\square$

## Graphical representation for the function $f(\zeta )$

Functions that operate on Complex numbers are referred to as Complex functions. An extension of the complex functions that accepts a complex number as input and returns a complex number is output. Input has two dimensions of information and output another two, giving us a total of four dimensions to fit into our graph. It is challenging to draw the graph for complex functions. Even though the Complex functions are often used for mapping and transformation, such as conformal mapping in complex analysis. The phase and absolute value diagrams help visualize how these mappings and transformations alter the complex plane, preserving angles or shapes, which is a fundamental property of conformal mappings. The conformal mappings find applications in engineering and physics, where complex numbers describe electrical circuits, waves, and quantum mechanics, among other things. Understanding the phase and magnitude of complex functions is essential for solving problems in these domains.

Phase and absolute value diagrams, also known as Argand diagrams or complex plane diagrams, are useful tools for visualizing and analyzing complex functions, whether they are univalent or multivalent. These diagrams help us understand the behavior of complex functions in terms of their magnitude (absolute value) and phase (argument) at various points in the complex plane. The phase diagram can help identify singularities (such as poles and branch points) as they typically manifest as discontinuities or infinite slopes in the diagram. The absolute value diagram can show the behavior of the function near these points, indicating if it approaches infinity or remains bounded.

**Figure 1:**
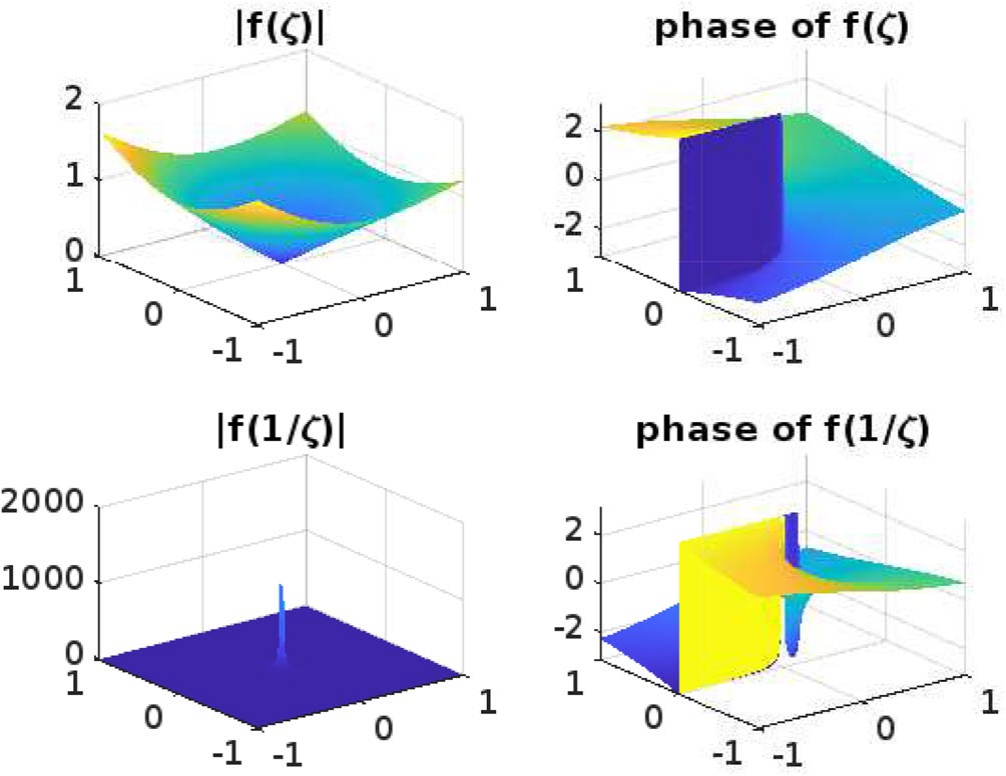
For $$\alpha =0.1; b=1; \nu =2; n=1; \delta =1; \lambda =1; p=1; \ \ $$ $$ -1\le Re (\zeta ) \le 1; -1\le Im (\zeta ) \le 1. $$

**Figure 2:**
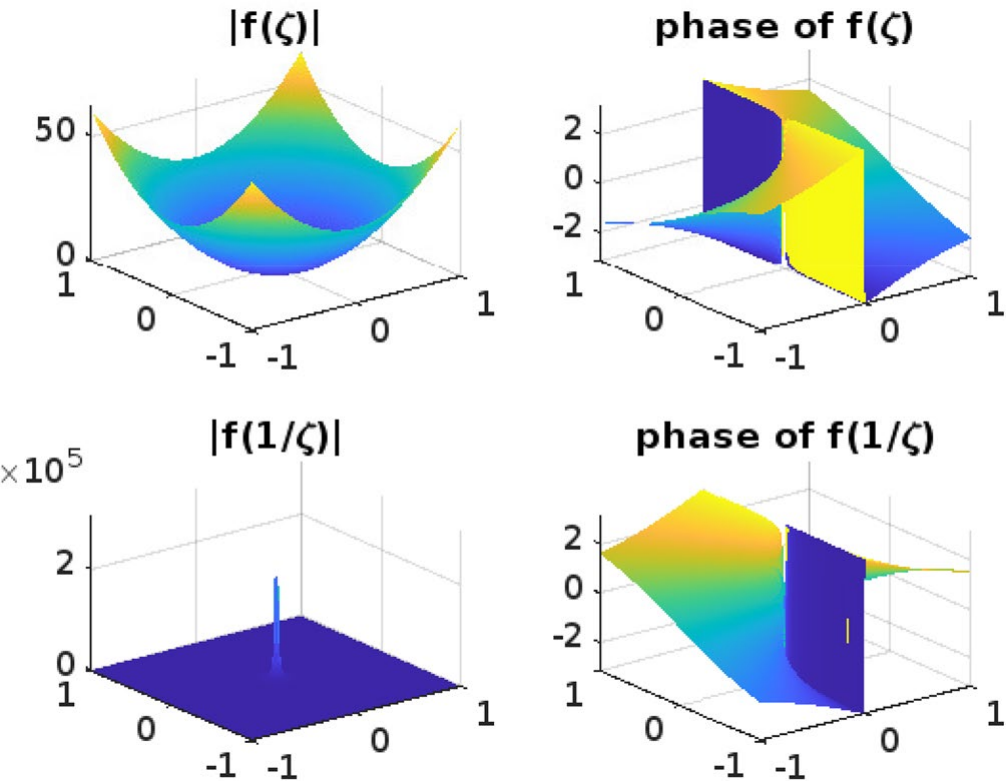
For $$\alpha =0.5; b=1; \nu =2; n=1; \delta =1; \lambda =1; p=10; \ \ $$ $$ -1\le Re (\zeta ) \le 1; -1\le Im (\zeta ) \le 1. $$

**Figure 3:**
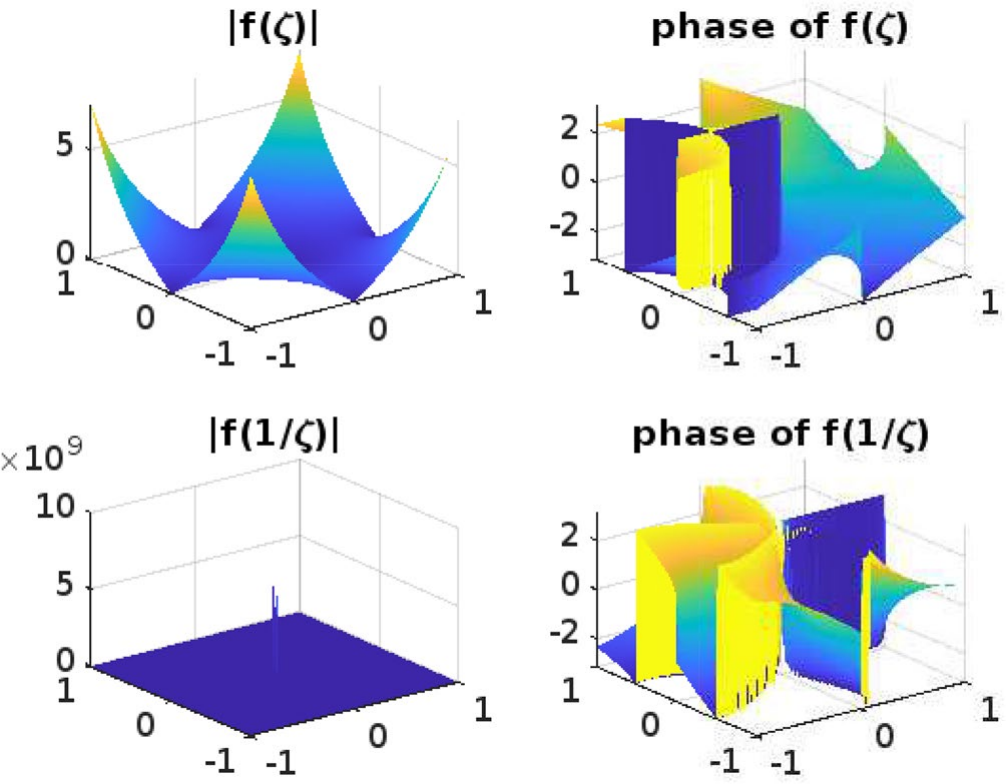
For $$\alpha =0.1; b=1; \nu =5; n=1; \delta =1; \lambda =1; p=5; \ \ $$ $$ -1\le Re (\zeta ) \le 1; -1\le Im (\zeta ) \le 1. $$

**Figure 4:**
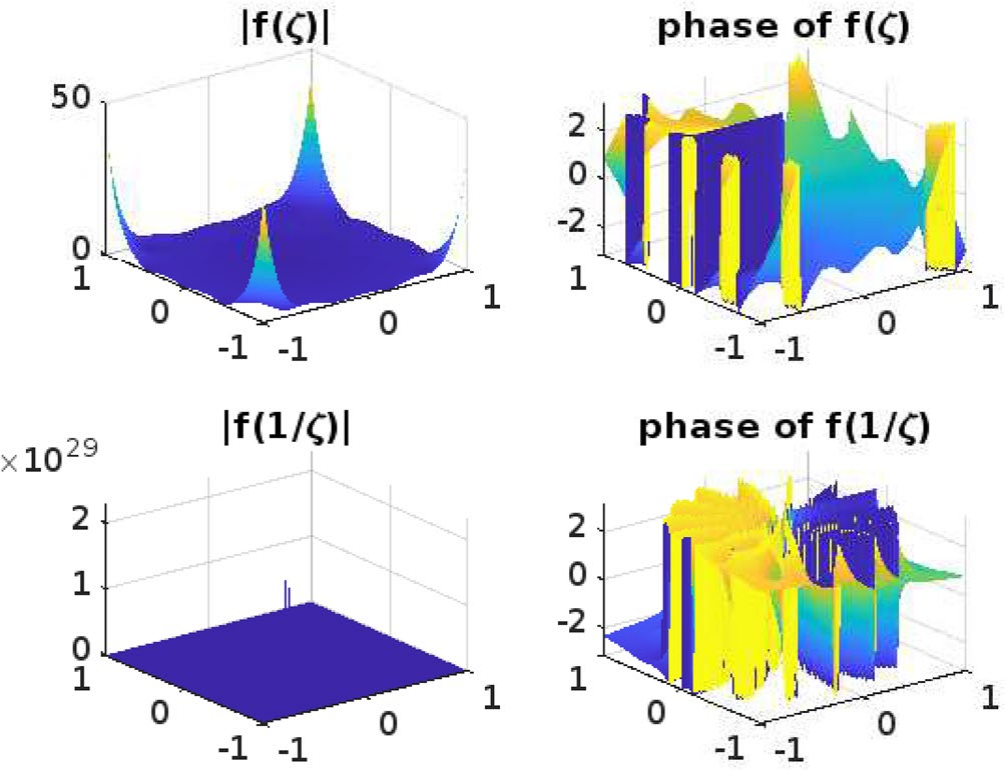
For $$\alpha =0.5; b=1; \nu =15; n=1; \delta =1; \lambda =1; p=10; \ \ $$ $$ -1\le Re (\zeta ) \le 1; -1\le Im (\zeta ) \le 1. $$

**Figure 5:**
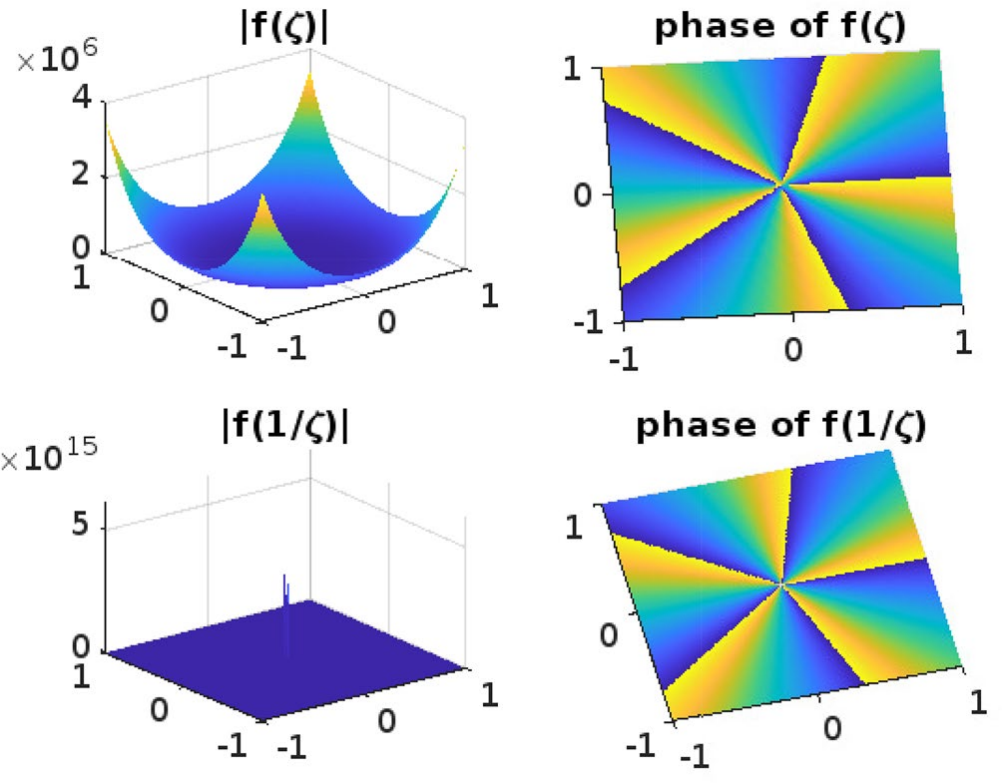
For $$\alpha =0.9; b=4; \nu =5; n=5; \delta =1; \lambda =1; p=20; \ \ $$ $$ -1\le Re (\zeta ) \le 1; -1\le Im (\zeta ) \le 1. $$

In this section, the phase and absolute values of the function$f(\zeta )$ from (11) have been examined (Figs. 1, 2, 3, 4 and 5) with various parameters and the following graphs (Figs. 1, 2, 3, 4 and 5) are drawn by using MATLAB. The phase and absolute values for the figures provide a geometric and intuitive way to understand the behavior of complex functions. They are particularly useful when dealing with univalent and multivalent functions, as they help identify key features, singularities, and transformations in the complex plane, making complex analysis more accessible and insightful.

## Conclusions

In this article, the coefficient inequality, extreme points, growth and distortion, starlikeness and convexity, and partial sums for a new subclass by using the linear operator have been examined. Furthermore, the graphs of extremal functions are analyzed in terms of how it has been expressed while replacing the suitable values of the parameters. This work motivates the researchers to extend the results of this article into some new subclasses of meromorphic functions and q-calculus.

## Data Availability

No datasets were generated or analysed during the current study.
